# Family-Based Study of Dermatoglyphic Patterns and Stress-Related Genetic Variability in Schizophrenia

**DOI:** 10.1192/j.eurpsy.2026.11265

**Published:** 2026-07-31

**Authors:** M. M. Moreira Martinez, N. Hostalet, A. Sotero-Moreno, C. Almodóvar-Payá, M. J. Muñoz, L. Fañanás, S. Miret, M. Giralt-López, M. Fatjó-Vilas

**Affiliations:** 1 Child and Adolescent Psychiatry Department, Germans Trias i Pujol University Hospital; 2 Germans Trias i Pujol Research Institute, Badalona; 3 Department of Psychiatry and Legal Medicine, Autonomous University of Barcelona, Cerdanyola del Vallès; 4 FIDMAG Germanes Hospitalàries Research Foundation, Barcelona; 5 CIBERSAM ((Biomedical Research Network in Mental Health; Instituto de Salud Carlos III), Madrid; 6 Department of Evolutionary Biology, Ecology and Environmental Sciences, Faculty of Biology, University of Barcelona, Barcelona; 7 Adolescent Psychiatry Unit, Fundación Hospitalarias Sant Boi, Sant Boi de Llobregat; 8 Adult Mental Health Centre, Psychiatry, Mental Health and Addictions Service, Santa Maria University Hospital, Lleida, Spain

## Abstract

**Introduction:**

Schizophrenia Spectrum Disorders (SSD) are neurodevelopmental conditions shaped by genetic and environmental factors during early brain development. Dermatoglyphic patterns can serve as markers of early developmental disruptions, with features such as simplified fingerprints and greater asymmetry observed more frequently in SSD patients and, to a lesser extent, in their unaffected relatives. Stress-regulation systems, particularly the corticotropin-releasing hormone (CRH) and endocannabinoid (ECS) pathways, are also implicated in SSD risk, yet their potential associations with dermatoglyphic variations remain largely unexplored.

**Objectives:**

To assess if variants in stress-related genes affect the transmission of dermatoglyphic traits in SSD.

**Methods:**

We studied 61 families (n = 208), including SSD patients and their unaffected relatives. Hand and finger dermatoglyphic patterns were assessed. Seven SNPs from Corticotropin-releasing hormone receptor 1 gene (CRHR1) and Cannabinoid Receptors type 1 and 2 *(CNR1*, *CNR2)* were genotyped. Family-based associations were tested using quantitative transmission disequilibrium tests (QTDT analysis/Unphased).

**Results:**

Genetic variants at *CRHR1* (rs17689966 and rs171440) and *CNR2* (rs2501431) were associated with the preferential transmission of higher finger and palmar asymmetric dermatoglyphic patterns from parents to affected offspring. The same pattern was detected for one *CRHR1* haplotype (rs171440–rs17689966) in relation to palmar dermatoglyphic ridge counts.

**Conclusions:**

Dermatoglyphic asymmetry may serve as a non-invasive marker of developmental instability linked to variations in stress-regulation genes, reinforcing the role of combined genetic and prenatal stress risk in SSD.

**Acknowledgements:**

ISCIII project-PI20/01002, grant-FI21/00093-NH, grant-CP20/00072-MFV, mobility grant-MV23/00059-NH, co-funded by ERDF/ESF; AGAUR: 2021SGR01475.

**Disclosure of Interest:**

None Declared

